# Dual-Functional Nanofibrous Patches for Accelerating Wound Healing

**DOI:** 10.3390/ijms231810983

**Published:** 2022-09-19

**Authors:** Dan Xia, Yuan Liu, Wuxiu Cao, Junwei Gao, Donghui Wang, Mengxia Lin, Chunyong Liang, Ning Li, Ruodan Xu

**Affiliations:** 1 Tianjin Key Laboratory of Materials Laminating Fabrication and Interface Control Technology, School of Materials Science and Engineering, Hebei University of Technology, Tianjin 300130, China; 2 Institute of Basic Theory for Chinese Medicine, China Academy of Chinese Medical Sciences, Beijing 100700, China

**Keywords:** coaxial electrospinning, wound healing, polycaprolactone, gelatin

## Abstract

Bacterial infections and inflammation are two main factors for delayed wound healing. Coaxial electrospinning nanofibrous patches, by co-loading and sequential co-delivering of anti-bacterial and anti-inflammation agents, are promising wound dressing for accelerating wound healing. Herein, curcumin (Cur) was loaded into the polycaprolactone (PCL) core, and broad-spectrum antibacterial tetracycline hydrochloride (TH) was loaded into gelatin (GEL) shell to prepare PCL-Cur/GEL-TH core-shell nanofiber membranes. The fibers showed a clear co-axial structure and good water absorption capacity, hydrophilicity and mechanical properties. In vitro drug release results showed sequential release of Cur and TH, in which the coaxial mat showed good antioxidant activity by DPPH test and excellent antibacterial activity was demonstrated by a disk diffusion method. The coaxial mats showed superior biocompatibility toward human immortalized keratinocytes. This study indicates a coaxial nanofiber membrane combining anti-bacterial and anti-inflammation agents has great potential as a wound dressing for promoting wound repair.

## 1. Introduction

Wound healing is a complex process involving hemostasis, inflammation, proliferation and remodeling, in which bacterial infections and excessive reactive oxygen species (ROS) production are two big roadblocks [1,2,3]. At the early stage of wound healing, the bacterial infection induced by microorganism accumulation hinders the healing process and even leads to the risk of mortality [4]. In addition, excessive ROS produced at the inflammation stage induces oxidative stress, lipid peroxidation and severe damage to cells, thereby delaying the transformation of the inflammation stage to the proliferation stage [5,6]. Therefore, the development of multi-functional wound dressings with both anti-bacterial activity and excessive ROS scavenging capacity is needed both in the academic studies and commercial applications [7].

Electrospun nanofibrous membranes show excellent performance as wound dressings [8,9,10], since they are able to provide a moist wound environment and load bioactive agents to promote wound healing. Their fibrous structure mimics the native extracellular matrix, permitting gas exchange and removing wound exudates, providing an ideal microenvironment for cell adhesion, proliferation, and further differentiation [11]. Moreover, coaxial electrospun nanofibers are capable of incorporating multiple bioactive agents in a single-step and delivering them at the same time under a controllable manner.

Polycaprolactone (PCL) is a prospective biomaterial for electrospinning because of its outstanding biocompatibility, mechanical properties, and easy electrospinning processing ability [12,13,14]. Electrospun PCL mats are often used in drug delivery systems to regulate fast drug release rate since they are extremely slow to degrade [15,16]. Gelatin (GEL) is one of the most popular polymers for electrospinning since it is biodegradable and biocompatible [17,18]. By coaxial electrospinning, a core-shell PCL/GEL nanofiber wound dressing can be prepared.

Several drug candidates have been used so far to manufacture wound dressings from electrospun fibers. As a well-known broad-spectrum antibiotic, tetracycline hydrochloride (TH) shows strong ability to enhance fibroblast adhesion and bacteriostatic activity against a variety of bacteria. As a highly water-soluble drug, TH loaded in a GEL shell could achieve massive release in the initial of skin repairing, thereby inhibiting bacterial infection [19,20]. Curcumin (Cur), a natural polyphenolic compound derived from turmeric [21], has been widely studied for its anti-inflammatory, antioxidant effects and low toxicity [22,23]. Applying Cur to wound healing can not only scavenge ROS, but also enhance cellular antioxidant capacity through the activation of the nuclear factor erythroid-2 related factor 2 pathway [24]. Meanwhile, Cur can also decrease the expression of pro-inflammatory cytokines, such as IL-6, and TNF-α [25]. In addition, Cur favors cell proliferation, migration, and collagen deposition, which can accelerate wound re-epithelialization in the later stages of wound healing [26]. However, due to the low bioavailability of Cur under physiological conditions, its application in wound healing is limited [27,28]. Therefore, loading Cur into the core layer of a coaxial drug carrier can improve its stability, achieving sustained drug release, and hence promote the skin regeneration.

**Scheme 1:**
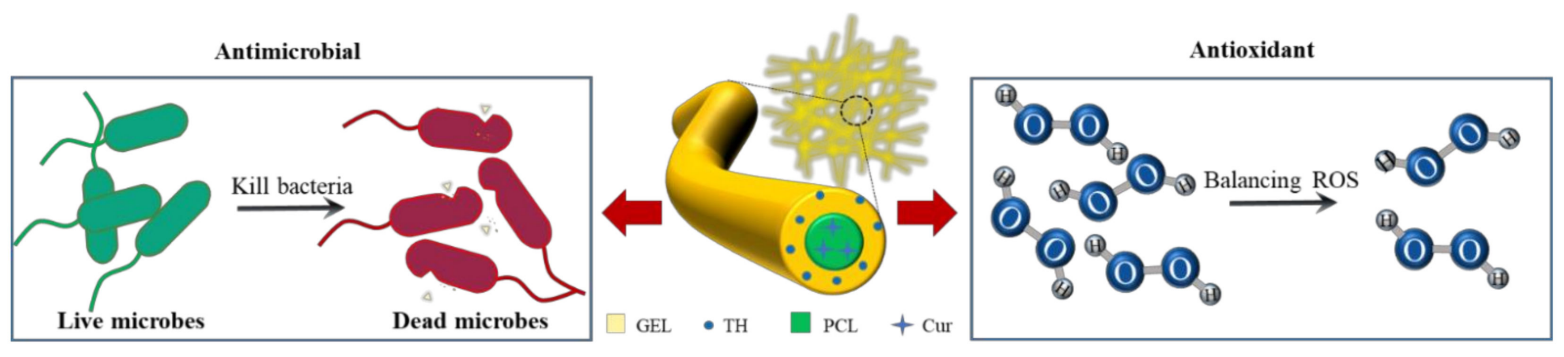
Dual-functional nanofiber patch with antibiotic TH and antioxidants Cur.

Herein, a coaxial PCL-Cur/GEL-TH nanofiber mat with the shell layer releasing TH for bacteriostasis, and the core layer releasing Cur for anti-inflammatory and anti-oxidative purposes, was prepared (Scheme 1). The physical and chemical properties of coaxial nanofibers, including surface morphology, hydrophilicity, water absorption, and mechanical properties, were characterized. In vitro drug release and antibacterial activity were also examined. In addition, the biocompatibility of the coaxial nanofiber mat was explored with human immortalized keratinocytes cells (HaCaT).

## 2. Results and Discussion

### 2.1. Nanostructure and Composition of Coaxial Nanofibers

**Figure 1:**
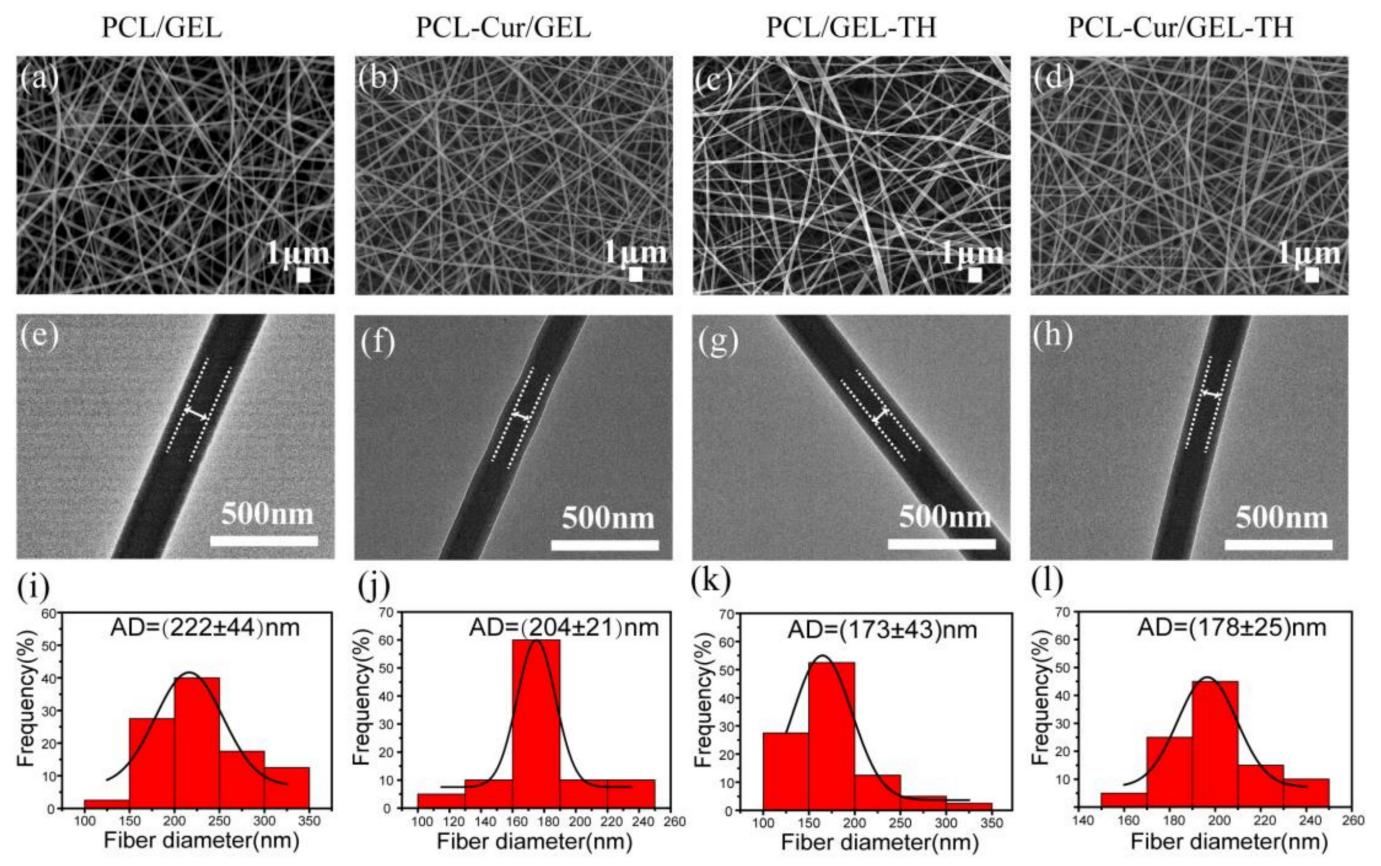
(**a**–**d**) SEM, (**e**–**h**) TEM images and (**i**–**l**) the corresponding diameter distribution of different coaxial nanofibers. The white dash line in (**e**–**h**) indicates the border between the PCL core and GEL shell.

Scanning electron microscopy (SEM) images (Figure 1a–d) showed that the nanostructures of coaxial nanofiber mats were distributed randomly and uniformly with smooth fiber surface, showing a large surface area and a distinct porous structure. The core-sheath structure of nanofibers was confirmed by transmission electron microscopy (TEM) characterization. The border between the PCL core and GEL shell can be seen in Figure 1e–h, in which drug loading did not affect the coaxial structures. The corresponding diameters of coaxial PCL/GEL, PCL-Cur/GEL, PCL/GEL-TH and PCL-Cur/GEL-TH nanofibers were 222 ± 44, 204 ± 21, 173 ± 43 and 178 ± 25 nm, respectively (Figure 1i–l). The drug-free PCL/GEL nanofiber had the largest diameter while the one and/or two drug(s) loaded nanaofibers show a somewhat smaller diameter. Numerous factors affect the size of electrospinning nanofibers, including solution properties such as viscosity, elasticity, electrical conductivity, and surface tension [29]. Only when in a proper range does the increase in conductivity favor the formation of finer fibers due to extensive bending of the jet. It has been reported that an effective strategy to increase the conductivity of electrospun polymer solutions is to introduce ionic compounds such as salts or inorganic compounds [29,30,31,32]. Therefore, the addition of TH and Cur may increase electrical conductivity if other parameters remain the same, thereby making the fibers thinner [30,33,34]. Coaxial nanofibers after glutaraldehyde crosslinking were also characterized (Figure S1). All the core/shell nanofibers became somewhat larger compared to before glutaraldehyde crosslinking, which may be caused by the moisture contained in the glutaraldehyde vapor leading to swelling and flattening of some nanofibers during the cross-linking process [35]. The SEM images of single GEL and PCL nanofibers are shown in Figure S2. Compared to coaxial nanofibers, the diameters of single PCL fibers were somewhat larger, while that of the single GEL fibers are smaller.

**Figure 2:**
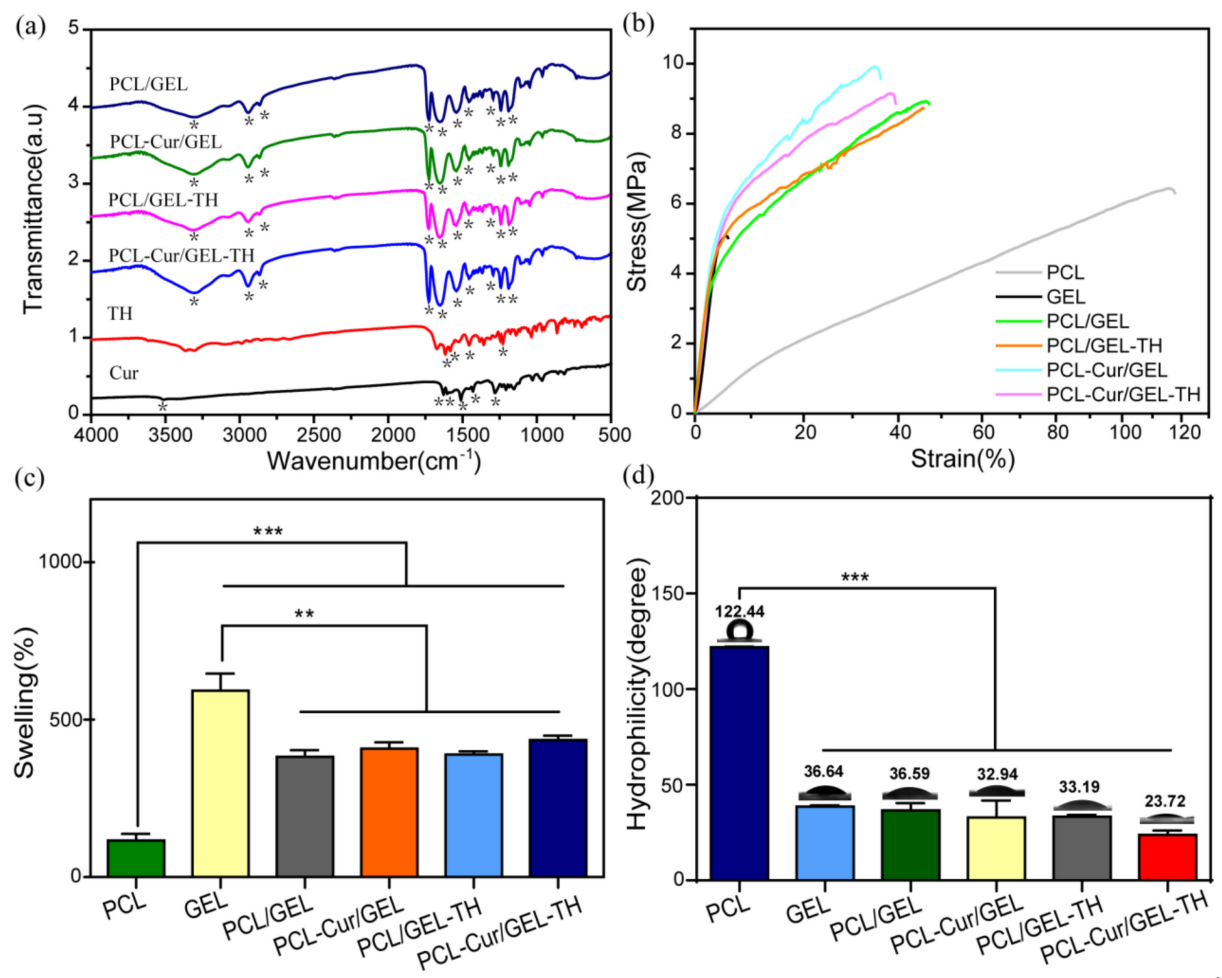
(**a**) FTIR spectra, the asterisks indicate the characteristic peaks of different samples. (**b**) Stress−strain curves. (**c**) Swelling abilities and (**d**) Water CAs (*n* = 3) of different nanofibrous membranes; * *p* < 0.05; ** *p* < 0.01; *** *p* < 0.001.

The chemical characteristics of different nanofiber mats were evaluated by Fourier transform infrared spectroscopy (FTIR) (Figure 2a). The characteristic peaks of different samples are summarized in Table S1. The results show that the characteristic peaks at 1647, 1548 ^1^, 2949 and 2865 cm^−1^ relate to the amide Ⅰ and amide Ⅱ bonds, asymmetric CH_2_ stretching and symmetric CH_2_ stretching of GEL, respectively [36,37]. The characteristic peaks at 1725, 1240 and 1165 (1191) cm^−1^ relate to the carbonyl group, C-O-C stretching, and symmetric C-O-C stretching of PCL [38,39]. All four core/shell nanofibers displayed both the feature peaks of PCL and that of GEL, verifying the successful incorporation of PCL and GEL in all core/shell nanofiber mats (Table S1). The feature peaks situated at 1430, 1510 and 1600 cm^−1^ in the Cur absorption curve are related to the bending vibration of C-H of olefin, the skeleton vibration of C-C of benzene ring and the stretching vibration of C=O, respectively. The characteristic peak at 1277 cm^−1^ is caused by the stretching vibration of C-O, while that at 3510 cm^−1^ is related to the phenolic hydroxyl group [40,41]. The characteristic peaks of TH (1613 and 1579 cm^−1^) relate to the C=O (A and C) ring and NH_2_ amide bond [42]. The feature peaks of three drug-loading coaxial nanofibers did not change compared to drug-free coaxial nanofiber. This may be caused by (1) the characteristic peaks of Cur, TH and PCL/GEL were overlapping, or (2) the drug dosages in three drug-loading coaxial nanofibers were low [43,44].

### 2.2. Mechanical Properties of Different Nanofiber Mats

The mechanical properties of wound dressings must be considered. For two-dimensional nanofibrous membranes, the tensile properties are the most important [45]. Tensile properties affect the degree of wound healing and human comfort, reflecting the dressing’s ability to adapt to human physique and movement. Since skin has a certain elasticity, when the dressing is easy to stretch and can return to its original position, it can prevent subcutaneous shear damage, the patient will not feel discomfort, and delayed wound healing caused by mechanical friction can be avoided [46]. Figure 2b shows the mechanical properties of different nanofiber mats. Detailed values of mechanical parameters are summarized in Table S2. The PCL nanofiber membrane showed a high elongation at break (115.53 ± 7.77%), indicating the strong toughness. The tensile strength and Young’s modulus of PCL nanofibers were 6.43 ± 0.14 MPa and 12.77 ± 0.02 MPa, respectively. In contrast, the elastic modulus of GEL was 118.66 ± 0.49 MPa, indicating the high brittleness of the GEL nanofiber. The mechanical properties of PCL/GEL coaxial nanofibers were between those of PCL and GEL, with a larger tensile strength (8.92 ± 0.49 MPa) compared with PCL and higher elongation (47.08 ± 5.03%) compared with GEL, which was caused by the addition of PCL and the core-shells in coaxial electrospinning protected each other to stabilize the jet [47], thereby producing better alignment and crystallization of the core layer material [48]. The mechanical properties of three drug-loading nanofibers mats were not much different from drug free nanofiber patch (PCL/GEL). Considering the final application of the nanofiber mats are in wet/hydrated condition. The mechanical properties of different nanofiber mats were tested in swollen conditions (Figure S3), which showed that the coaxial scaffolds still had good tensile properties in the hydrated state. The elongation at break of the double drug-loaded coaxial scaffold (PCL-Cur/GEL-TH) was comparable to that of human skin (60–75%), meeting the requirements of wound dressings [7]. The high tensile strength and elongation at break indicate that the mechanical properties of PCL-Cur/GEL-TH can satisfy the needs of wound dressings [49].

### 2.3. Water Absorption Ability of Coaxial Nanofibers

Nanofiber dressing with good water absorption capacity results in good ability to absorb wound exudate [50]. The swelling capacity of different samples is shown in Figure 2c. It is apparent that the water absorption rate of single GEL is the highest (592.53%), which is due to the amine and carboxyl functional groups of GEL [51], enabling GEL to absorb five to ten times more water. The swelling behavior of single PCL was the lowest (116.97%). The water absorption capacity of all core/shell nanofibers was between that of single GEL and single PCL. The corresponding water absorption rate (*AR_water_*) of PCL/GEL, PCL-Cur/GEL, PCL/GEL-TH and PCL-Cur/GEL-TH were 382.62, 408.30, 389.76 and 435.00%, respectively. This is because the outer layer of GEL increases the *AR_water_*, but the hydrophobic inner layer of PCL lowers the numerical value. In addition, the amount of drug loading had little effect on the *AR_water_* with no significant difference between the *AR_water_* of the fiber membranes with dual drug loading or single drug loading. The swelling trend from 0 to 7 days was monitored to check the stability of different samples at 37 °C (Figure S4). The water absorption ability of PCL was kept at a low level. The water absorption rate of GEL reached the maximum at day one (554.07%), and then began to decrease at day two because of the degradation of GEL leading to the loss of quality of GEL. Due to the addition of PCL, all the coaxial scaffolds showed good water adsorption stability, maintaining good water absorption performance.

A wound dressing must keep the wound moist [33]. High water absorption means that the wound dressing has good ability to absorb wound exudate and keep the wound moist, which is conducive to the growth of granulation tissue and prevents scarring. On the contrary, dry environment cause wound dehydration and the granulation tissue spreads slowly, delaying wound healing and ultimately resulting in scarring. Compared with single PCL, the coaxial dressing had good water absorption, since the addition of GEL greatly improved the water absorption of PCL, which is conducive to wound healing. It was reported by Chen et al. [52] that a novel CUR-loaded sandwich-like nanofibrous membrane (CSNM) with comparable water absorption (450%) to our samples was good for wound healing. Zou et al. reported that the swelling rate of PVA/CS nanofibrous membrane loaded with different concentrations of OH-30 nanoparticles reached the highest water absorption ability (about 450%) in several hours, which promoted wound healing [53]. Therefore, the superb *AR_water_* with the core/shell fibrous membrane is more suitable as a wound dressing.

### 2.4. Hydrophilicity of Core-Shell Nanofibers

The hydrophilicity of different fiber membranes was assessed by measuring their water contact angles (CAs) (Figure 2d). The results show the average CA of PCL nanofiber membrane was 122.44°, which is not conducive for wound healing [52]. However, it can be improved by incorporation of the hydrophilic GEL with a CA of 36.64°. By incorporating GEL with PCL, the CA of core/shell PCL/GEL was reduced to 36.59°. The addition of Cur and/or TH into the nanofibers further reduced the CA of the core/shell nanofibers since the addition of the drug(s) increased the charge density of the jet surface, resulting in greater draft under the electric field and thinner fibers. Fibers with small diameter possess large specific surface area, which will allow for more interfacial activity, favoring water-surface contact [54]. For coaxial nanofibers, the outer shell is GEL, which is hydrophilic. Therefore, the smaller diameter leads to smaller CA according to the Wenzel model, which concludes that the greater the roughness, the more hydrophilicity for hydrophilic surfaces and the greater the roughness, the more hydrophobicity for hydrophobic surfaces [54,55,56].

### 2.5. In Vitro Drug Release from Coaxial Nanofibers

**Figure 3:**
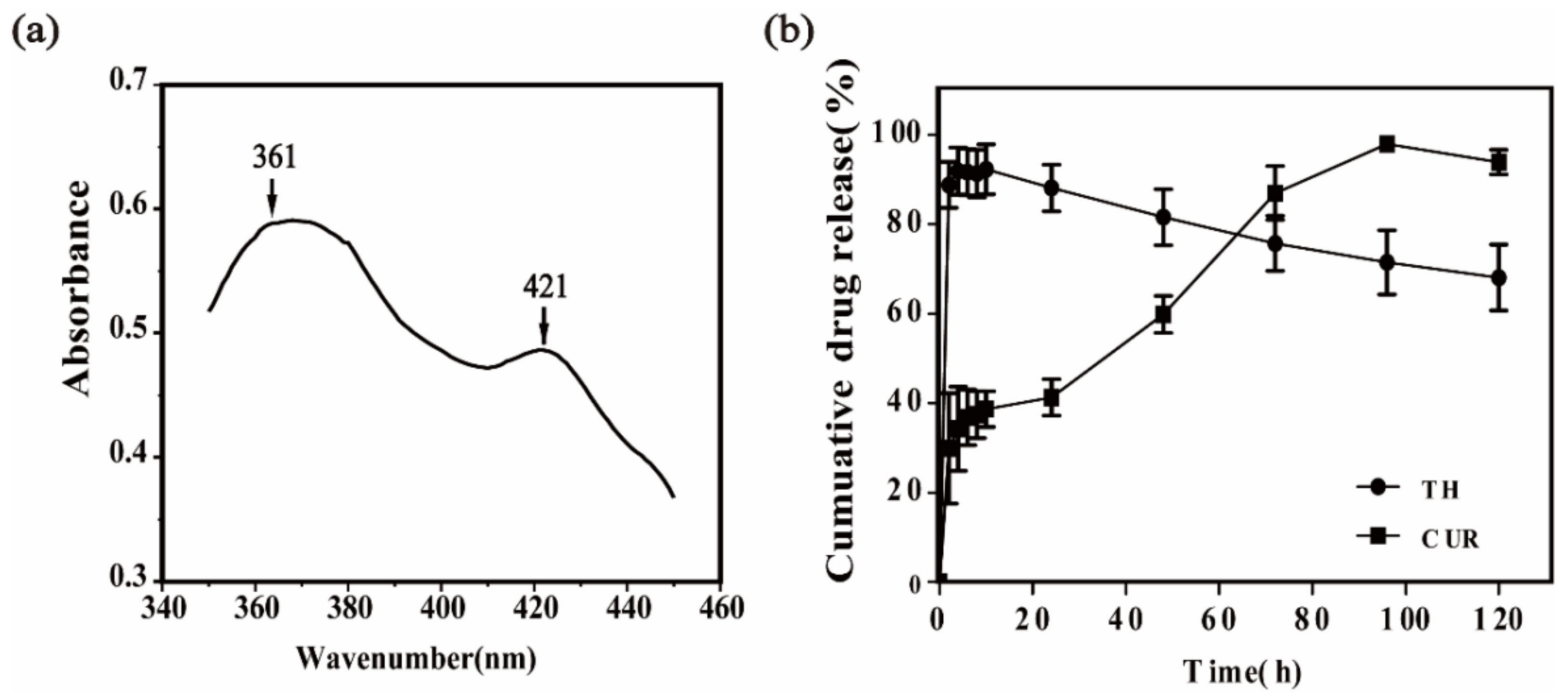
(**a**) Absorbance of CUR and TH at different wavelengths. (**b**) Drug release curves of PCL-CUR/GEL-TH.

The key to controlling drug release rate lies in the physical and chemical properties of the carrier material and the essential properties of the drug. The main factors include the swelling and degradation properties of the carrier material, the interaction between the material and the drug molecule, the size and distribution of the carrier, and the drug concentration difference in solution [57]. The absorbance of CUR and TH at different wavelengths (Figure 3a) and in vitro release profiles of Cur and TH (Figure 3b) tested in PBS (pH = 7.4) at 37 °C are shown in Figure 3. The results show that both Cur and TH had a burst release phenomenon during the initial 2 h, reaching release rates of 29.87% and 88.79%, respectively. This is because the drug release from nanofibers is mainly based on diffusion, from high drug concentration to low concentration. When the drug-loaded film was just placed in PBS, there was no drug in the PBS, which caused the drug to diffuse into PBS. Drug enrichment on the nanofiber surface usually led to undesired burst release [57]. The subsequent release of both drugs slowed down, with TH reaching the highest release rate of 92.25% in about 10 h. This is because the outer layer of GEL absorbs water and swells rapidly, resulting in a loose structure and rapid outward diffusion of the drug. The release rate of Cur at 24 h was 41.26%, and then reached the highest release rate of 97.89% at 98 h, with a slow and long-term release manner. This is because CUR is a hydrophobic drug with low solubility, and the drug moves with difficulty in the polymer. Thus, the degradation of the polymer is the controlling factor for drug release [38]. The drug on the surface can be released smoothly, while the drug on the inside must reach the surface before it is released. The release rate of the drug decreases with increasing distance of drug diffusion and migration to the surface [57]. Therefore, CUR release must pass through the core layer and then through the shell layer. The in vitro drug release profiles of PCL/GEL-TH and PCL-Cur/GEL were also measured (Figure S5), and showed similar trends to that of PCL-Cur/GEL-TH. This result is consistent with that reported previously. Ramalingam et al. studied the drug release rates of PCL/GEL blends, in which the shell required 4.9 h to achieve 50% release of the extracts while core layer needed 2.1 and 3.3 days to release of the extracts, indicating sustained release of drugs from core nanofibers [33]. Li et al. reported a DLS double-layer scaffold achieving the rapid release of LID (about 85%) in 6 h and sustained release of mupirocin for 5 days [39].Therefore, the PCL-Cur/GEL-TH coaxial nanofiber membrane can realize the sequential delivery of TH and Cur, in which the rapid release of TH can meet the antibacterial needs in the early stage of wounds, and the sustained release of Cur can meet the anti-inflammatory and antioxidant needs during the inflammation stage of wound healing.

### 2.6. Antibacterial Activity of Different Nanofibers

**Figure 4:**
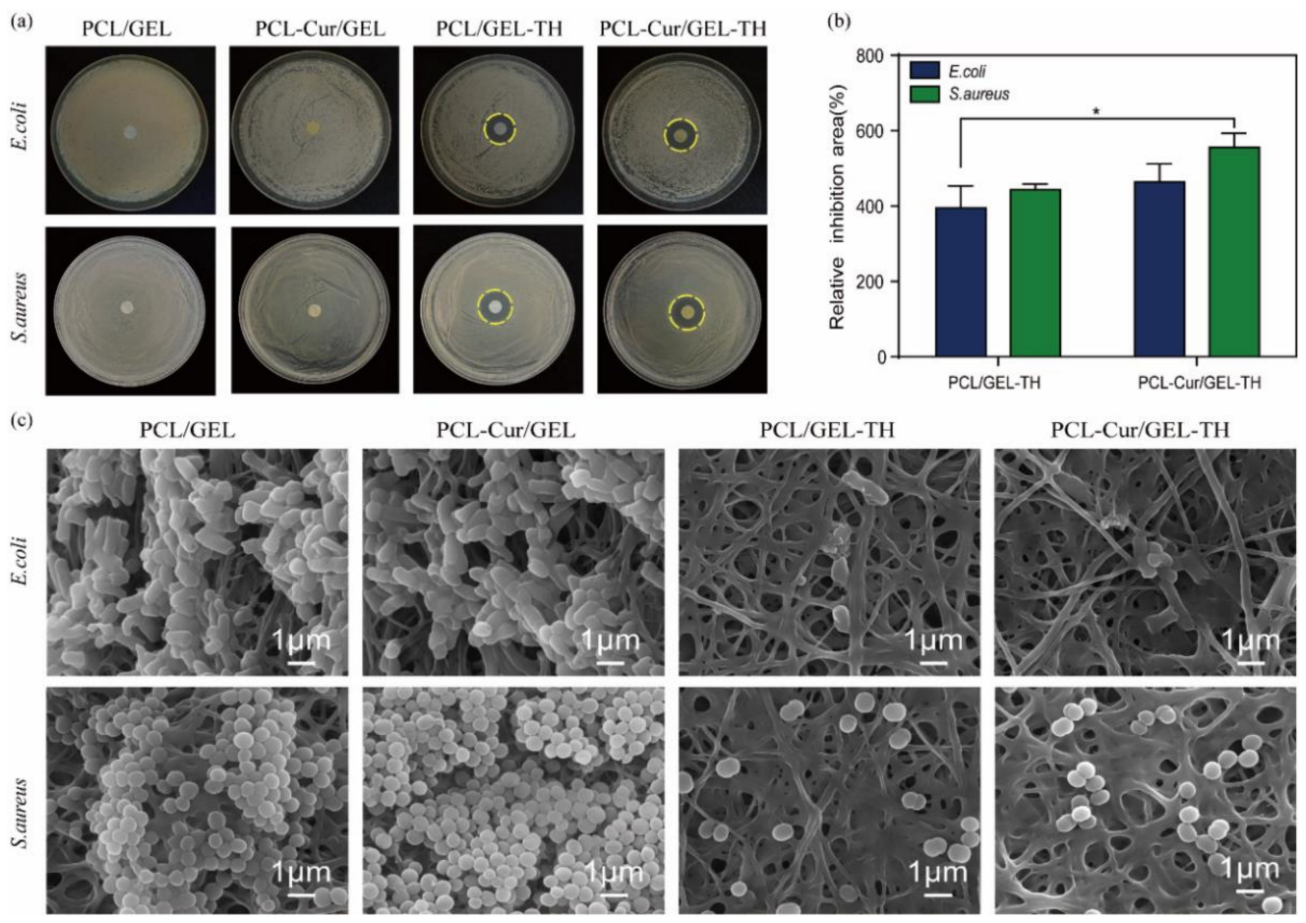
Antibacterial activity of different samples against *E. coli* and *S. aureus*. (**a**) Inhibition zone analysis. The yellow dotted circle in the figure is the inhibition zone. (**b**) Inhibition area of different samples (*n* = 3). (**c**) SEM images of *E. coli* and *S. aureus* on different membranes. * *p* < 0.05.

Inflammation in medicine refers to the defensive response of living tissue with vascular systems to damaging factors, and is caused by many factors such as bacteria, trauma, thermal injury, and chemical injury. Infection is a type of inflammation caused by bacteria or other microorganisms. Excessive inflammatory responses from bacterial infection can cause wounds difficult to heal [58]. Therefore, it is essential for wound dressings to possess anti-bacterial properties. The anti-bacterial properties of different nanofibrous membranes were assessed to quantify the inhibition zone by using the disk diffusion method (Figure 4a). The PCL/GEL and PCL-Cur/GEL coaxial nanofiber patches showed no obvious antibacterial activity since the inhibition zone was almost invisible. However, the inhibition diameters of PCL/GEL-TH and PCL-Cur/GEL-TH against *Escherichia coli* (*E. coli*) were 17.79 ± 1.05 mm and 18.42 ± 0.26 mm, while those against *Staphylococcus aureus* (*S. aureus*) were 18.98 ± 0.82 mm and 20.48 ± 0.58 mm, respectively. The inhibition zone value of PCL-Cur/GEL-TH was close to that of PCL/GEL-TH nanofibers on *E. coli* and *S. aureus*. Since the rapid release of the antibacterial drug TH occurred at the shell, an obvious inhibition zone was found. However, the inhibition zone value of PCL-Cur/GEL-TH was similar to that of PCL/GEL-TH, because Cur has little antibacterial effect and the antibacterial effect came from the TH. Therefore, the inhibition zone value of PCL-Cur/GEL-TH and PCL/GEL-TH nanofibers on *E. coli* and *S. aureus* were not significantly different. The relative inhibition areas of PCL/GEL-TH and PCL-Cur/GEL-TH against *E. coli* were 395.10 ± 58.56 and 463.38 ± 48.13%, while those against *S. aureus* were 443.35 ± 14.94 and 555.62 ± 37.11%, respectively (Figure 4b). The inhibition area results showed no significant difference in the inhibitory effects of PCL/GEL-TH and PCL-Cur/GEL-TH nanofibers on *E. coli* or *S. aureus*, since the same amount of TH were contained in both samples. In addition, the inhibitory effects of PCL/GEL-TH or PCL-Cur/GEL-TH on *E. coli* and *S. aureus* bacteria were also similar. These results were also verified by SEM images (Figure 4c). From the SEM results, one can observe that bacteria adhered to the PCL/GEL and PCL-Cur/GEL nanofibrous membranes grew uninterruptedly, whereas the bacteria on PCL/GEL-TH and PCL-Cur/GEL-TH membranes were much less because the released TH killed the bacteria. Bacteria killed by antibiotics lost their adhesion to the substrate, while the live bacteria still adhered to the membrane [59,60]. The results indicated that the bacteriostatic effect of Cur is not obvious but that of TH is significant. These results are consistent with the literature reported previously, in that Ramalingam, et al. used the same method to verify the antibacterial activity of the USE/Mino nanofibers and concluded that the prepared nanofibers inhibited bacterial adhesion and biofilm formation onto the nanofiber surfaces [33].

### 2.7. Antioxidant Activity of Coaxial Nanofibers

**Figure 5:**
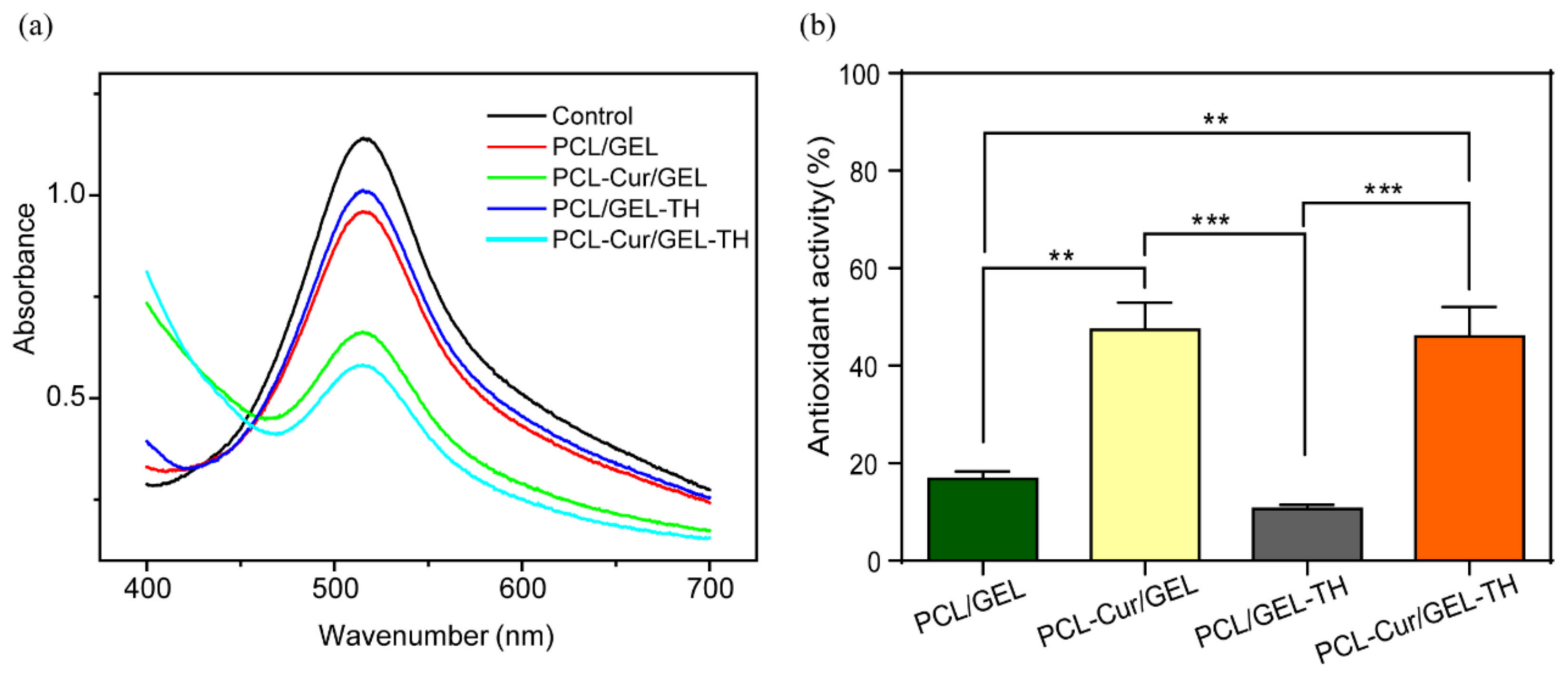
(**a**) Absorbance of DPPH solution with different nanofibrous membranes. (**b**) DPPH scavenging activity of different nanofiber membranes. ** *p* < 0.01; *** *p* < 0.001.

Numerous studies have proved that applying antioxidants during the wound healing process can significantly accelerate wound healing [61]. Cur displays antioxidant activity and plays a significant biochemical role in the process of removing reactive oxygen species from wounds and inhibiting lipid peroxidation [62]. To explore whether Cur released from coaxial nanofiber membranes could effectively scavenge ROS, DPPH radical scavenging experiments were performed. DPPH shows maximum absorbance at 517 nm. Absorbance changes of different samples after mixing with DPPH solutions for certain period of time were measured to evaluate the antioxidant activity of different samples (Figure 5). This showed that the control group had the highest absorbance while the absorbance of all experimental groups decreased slightly compared with the control group, among which the absorbance of PCL-Cur/GEL and PCL-Cur/GEL-TH groups decreased dramatically (Figure 5a). The DPPH radical scavenging activities of different samples are shown in Figure 5b. The results show that the DPPH free radical scavenging rate (*FRSR*) of PCL/GEL and PCL/GEL-TH were 16.83% and 10.59% while that of PCL-Cur/GEL and PCL-Cur/GEL-TH were 47.37% and 45.93%. Cur only released ~10% at 30 min (Figure 3), and the outstanding antioxidant activity of PCL-Cur/GEL and PCL-Cur/GEL-TH indicated a stronger scavenging effect of Cur on DPPH free radicals.

### 2.8. Cytotoxicity of Different Nanofibrous Membranes

**Figure 6:**
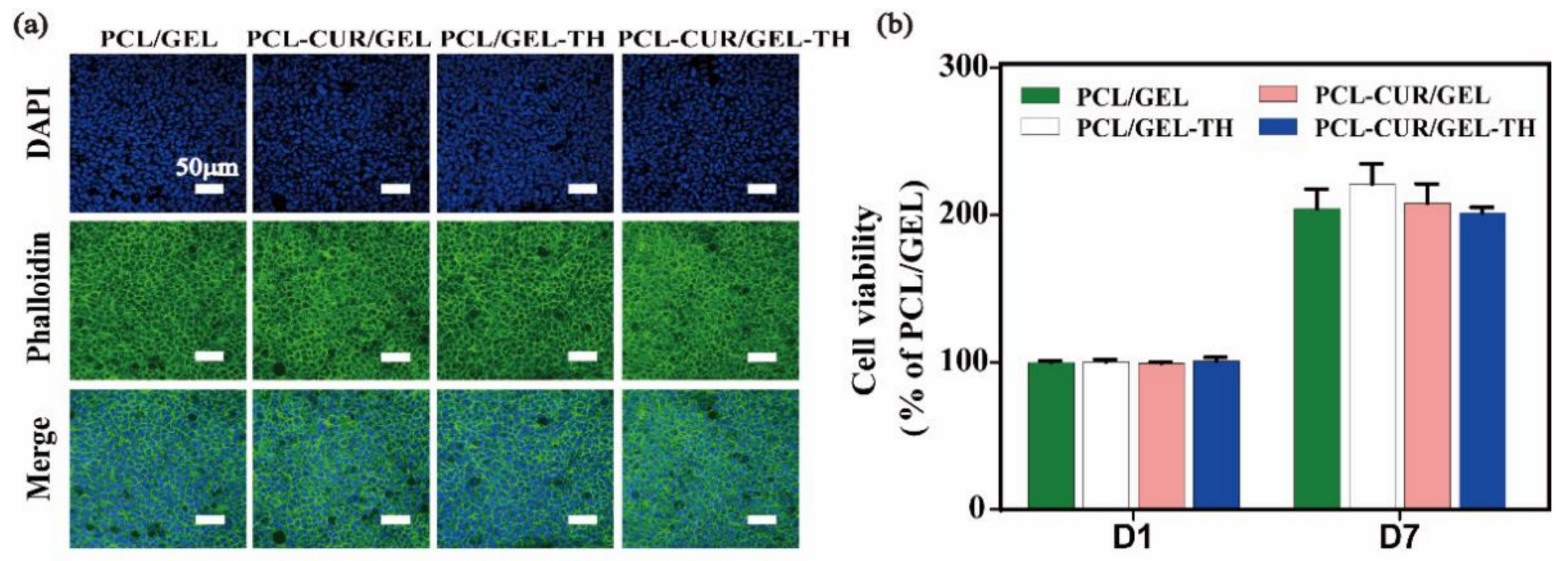
(**a**) Representative images from immunofluorescence staining of HaCaT cells on four different nanofiber membranes after 7-day cell culture. The F-actin cytoskeleton was labeled as green by Alexa Fluor 488 Phalloidin, while the nucleus was labeled as blue by DAPI. (**b**) Cytotoxicity effects of four different coaxial nanofiber mats on human HaCaT cells.

An ideal wound dressing should have good biocompatibility [63]. Figure 6 shows the biocompatibility of various coaxial nanofibers towards HaCaT cells. The representative images from immunofluorescence staining of samples (Figure 6a) showed the cell morphological structures on four coaxial nanofiber membranes were similar, indicating no obvious effect of different nanofibers on the growth of HaCaT cells. All four nanofibrous mats had good biocompatibility regardless of whether Cur or/and TH was loaded. The cell viability of HaCaT on different coaxial nanofiber membranes were shown in Figure 6b. The results showed that the cell viability of each group of samples was close to 100% at day 1. However, the cell proliferation on day 7 was only about two times that of day 1. This was because the number of cells seeding on the fiber was too high, and the thickness of fiber membrane used in this study was relatively thin, so contact inhibition enabled cells to cease proliferation when they contacted each other. It was also observed that HaCat cells grow well on the surface of fibers. Therefore, the prepared dual drug-loaded PCL-Cur/GEL-TH nanofibrous membrane can be applied for wound repairing.

## 3. Materials and Methods

### 3.1. Materials

PCL with an average Mn of 80,000 and GEL derived from porcine skin (Type A) were purchased from Sigma-Aldrich (St. Louis, MO, USA). 2, 2, 2-Trifluoroethanol (TFEA) was obtained from Macklin Biochemical Technology Co., Ltd. (Shanghai, China). Cur and TH were supplied from Aladdin Reagent Co., Ltd. (Shanghai, China). Glutaraldehyde was bought from Fuchen chemical reagent Co., Ltd. (Tianjin, China). Human immortalized keratinocytes cells were bought from Hyclone Co. Ltd. (Logan, UT, USA).

### 3.2. Preparation of Coaxial Nanofibers

The PCL solution with concentration of 9% (*w/v*) was made by dissolving 0.45 g PCL in 5 mL TFEA while the solution of GEL (10% *w/v*) was made by dissolving 1 g GEL in 10 mL deionized (DI) water and TFEA (1:3 *v/v*). Both solutions were gently stirred overnight at room temperature (RT). A coaxial nozzle composed of an inner 21-gauge needle with an inner diameter of 0.51 mm and an outer 15-gauge needle with an inner diameter of 1.37 mm was used for coaxial electrospinning with an applied voltage of 8.5 kV to prepare PCL/GEL membrane. The receiving distance was set to 15 cm while the flow rate of shell and core solution was set as 0.004 mL/min and 0.002 mL/min, respectively. For preparing drug-loaded membranes (PCL-Cur/GEL, PCL/GEL-TH and PCL-Cur/GEL-TH), the Cur and/or TH (1% *w/w*) were/was dissolved into the PCL and/or GEL solution(s) and stirred sufficiently. The drug-loading core/shell membranes were fabricated using the same method as that of PCL/GEL [64]. Glutaraldehyde (2.5% *w/w*) was used to cross-link GEL and all coaxial nanofibers for improving their stability in solution. The glutaraldehyde solution (2.5% *w/w*) was prepared by mixing 1 mL of DI water and 19 mL of glutaraldehyde (50% *w/w*). Crosslinking was carried out in a desiccator containing 20 mL of 2.5% *w/w* glutaraldehyde. The nanofiber membranes were cross-linked under glutaraldehyde vapor at 25 °C for 1 h, and then dried at room temperature for 3 h to eliminate glutaraldehyde residues [42,65].The single PCL and GEL nanofiber membranes were prepared for comparison. The detailed parameters were shown in Table S3.

### 3.3. Characterization of Different Nanofibers

Nanofiber morphology was observed by SEM (JSM-7100F, JEOL, Japan, 10 kV) after the membranes were coated by gold. The diameter distribution was quantitatively analyzed on SEM images by Image-Pro Plus with at least 50 diameters randomly selected. The core-shell structure of the nanofibers was verified by TEM (Talos F200S, FEI, USA). The chemical composition of nanofibers was examined by transmission FTIR (TENSOR 27, BRUKER, German).

The mechanical properties of the nanofiber membranes (1 cm × 4 cm) in the dry and hydrated state were tested with a load of 20 N (HY-0580) and a tensile speed of 5 mm/min for tensile properties test at room temperature. Three replicates were made for each sample.

*AR_water_* was characterized by soaking the samples (10 mm × 10 mm) in PBS (pH = 7.4) and then weighted instantly followed by eliminating the water on the sample surface. The *AR_water_* was calculated by the equation of *AR_water_* = (*M* − *M0*)/*M0* × 100%, where *M0* and *M* are the nanofibers of dry and wet weight, respectively.

The hydrophilicity of the nanofiber membranes was measured by the solid drop method (JC2000DM). The CA was measured after a droplet of DI water (3 μL) was immobilized freely onto the surface of a square nanofiber membrane (1 cm^2^). Considering the hydrophilicity of the GEL shell, the CA values were recorded immediately and quickly after the DI water dropped freely onto the surface. In addition, according to the Wenzel model, the surface roughness affects the CA. Therefore, the CA was tested on different spots of the same nanofiber membranes and average values recorded.

### 3.4. In Vitro Drug Release

The drug release profiles were tested by immersing drug-loaded membranes (20 mg) in PBS (10 mL) containing 0.5% Tween-80 as the dissolution medium. The samples were incubated at 37 °C and shaken constantly with a speed of 100 rpm. At presetting time points (2, 4, 6, 8, 10, 24, 48, 72, 96 and 120 h for Cur and TH), the absorbance of drug-contained solutions was measured by UV-visible spectroscopy under the wavelength of 361 nm (TH) and 421 nm (Cur). Three replicates were measured for each sample. After that, fresh PBS solution (3 mL) was added for maintaining the same environment for drug-release. A standard curve was plotted to calculate the density and cumulative releasing of the drugs and the release rate was related to the drug amount added initially. Then, the in vitro drug release curve versus time was plotted.

### 3.5. Antibacterial Activity

Antibacterial activity of nanofibrous mats against Gram-positive *S. aureus* and Gram-negative *E. coli* was determined by the disk diffusion method. The inhibition zone was determined by incubating different nanofiber plates with a diameter of 8 mm with bacteria (10^6^ CFU/mL) on agar plates at 37 °C for 12 h. The inhibition area (*IA*) was calculated by the equation of *IA* (%) = (*S* − *S0*)/*S0* × 100, where *S* and *S0* are the area of inhibition zone and nanofiber mat, respectively. Each characterization was duplicated three times.

SEM observation. Different samples (8 mm diameter discs, after UV sterilization) were placed in a 96-well plate, and 100 μL of bacteria with a concentration of 10^7^ CFU/mL were dropped into each well plate containing the samples and cultured in a constant temperature incubator for 24 h. Then the bacterial solution was sucked out, rinsed with PBS, 2.5% glutaraldehyde fixative was added, and placed in a 4 °C refrigerator overnight. The fiber membranes were dehydrated for 10 min using 30, 50, 70, 80, 85, 90, 95 and 100% ethanol, respectively. The fiber membranes after dehydration were air-dried naturally, and the morphology of bacteria was observed by SEM after gold spraying.

### 3.6. Antioxidant Activity

Four kinds of nanofiber membranes (15 mg) were placed in a centrifuge tube (4 mL), and then DPPH solutions (3 mL, 1 × 10^−4^ mol/L) were added. The DPPH solution without film was the control. After being stabilized in a dark environment for 30 min, the absorbance of each group at 517 nm wavelength was measured by UV-Vis, and the *FRSR* of each group was obtained by the equation: *FRSR* (%) = (*A0* − *A*)/*A0* × 100, where *A*_0_ is the blank absorbance and *A* is the absorbance of the samples. Three replicates were made for each sample.

### 3.7. Cell Culture

Both sides of different samples of 8 mm diameter were irradiated under UV light for 30 min. Hacat cells at a density of ~9×10^4^ were seeded onto 48-well microplates and cultured for 24 h and 7 days in medium with high glucose DMEM (Gibco) complemented with L-glutamate and sodium pyruvate supplements with 10% FBS (Gibco) in a humidified atmosphere at 37 °C and 5% CO_2_. Three specimens for each type of mat were tested.

### 3.8. In Vitro Cytotoxicity Evaluation

Viability was tested by using the CellTiter96® Aqueous One Solution Cell Proliferation Assay Kit (Promega, Madison, WI, USA). Briefly, the medium was removed after 24 h of culture, and four sets of scaffolds were immersed in each well of diluted MTS solution and cultured for 4 h. Medium with same volume and cell-free MTS reagent was also incubated as background. After transferring the solution (100 µL) to a 96-well plate, the absorbance of each well at 490 nm was tested.

### 3.9. Immunocytochemistry

The morphology of cells cultured for 24 h was characterized by confocal fluorescence microscopy (Olympus FV3000). First, each sample was fixed with 4% paraformaldehyde (10 min, RT) and rinsed with PBS 3 times. Second, a permeabilization solution containing 0.2% Triton X-100 was added into the samples (10 min) and rinsed with fresh PBS (3 times) afterwards. Third, nanofiber samples with cells on were blocked with 1% BSA in PBS (1 h, RT). The F-actin and nuclei of cells were immunostained with Alexa Fluor 488 Phalloidin (Thermo Fisher, Waltham, MA, USA) and DAPI (Sigma, St. Louis, MO, USA), respectively.

### 3.10. Statistics

All quantitative results are represented as the means ± standard deviation. Student’s *t*-test was used to analyze different data groups statistically. The *p* < 0.05 indicates significantly difference statistically.

## 4. Conclusions

In summary, a dual functional nanofiber wound dressing (PCL-Cur/GEL-TH) was successfully prepared by loading the anti-bacterial agent TH in the shell layer and the anti-inflammatory agent Cur in the core layer of coaxial PCL/GEL nanofibers. The coaxial PCL-Cur/GEL-TH nanofibers membranes showed good water absorption, surface hydrophilicity and mechanical properties. Drug-releasing results showed the PCL-Cur/GEL-TH enable rapidly released TH with a cumulative release rate of 92.25% within 10 h, and sustained release of Cur with a cumulative release rate of 97.89% at 98 h, maintaining anti-inflammatory and antioxidant effects. In vitro antibacterial experiments show that this had inhibitory effects on both Gram-negative and Gram-positive bacteria. PCL-Cur/GEL-TH also showed strong scavenging effects on free radicals with an antioxidant activity of 45.93%. In addition, the PCL-Cur/GEL-TH coaxial nanofiber membranes showed good biocompatibility towards HaCaT cells. The prepared PCL-Cur/GEL-TH could be a promising dual-functional dressings for wound healing.

## Data Availability

The authors declare that all data supporting the findings are available within the paper or are available from the authors upon request.

## Supplementary Materials

The supporting information can be downloaded at: https://www.mdpi.com/article/10.3390/ijms231810983/s1.
