# Supplementary material for: Dual-Functional Nanofibrous Patches for Accelerating Wound Healing
Source: Int J Mol Sci. 2022 Sep 19;23(18):10983. doi: 10.3390/ijms231810983 (PMC9502447; doi:10.3390/ijms231810983)
Supplement: Supplementary file 1 [file ijms-23-10983-s001.zip › ijms-1861168-supplementary.pdf]

Supplementary Materials

Figures

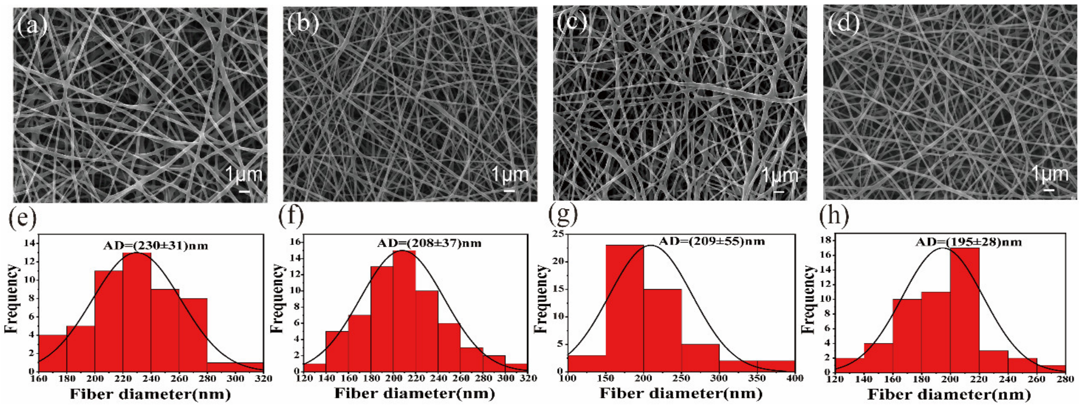

Figure S1. (**a**–**d**) SEM images and (**e**–**h**) corresponding diameter distribution of different coaxial nanofibers after the glutaraldehyde crosslinking; (**a**,**e**) PCL/GEL, (**b**,**f**) PCL-CUR/GEL, (**c**,**g**) PCL/GEL-TH, (**d**,**h**) PCL-CUR/GEL-TH.

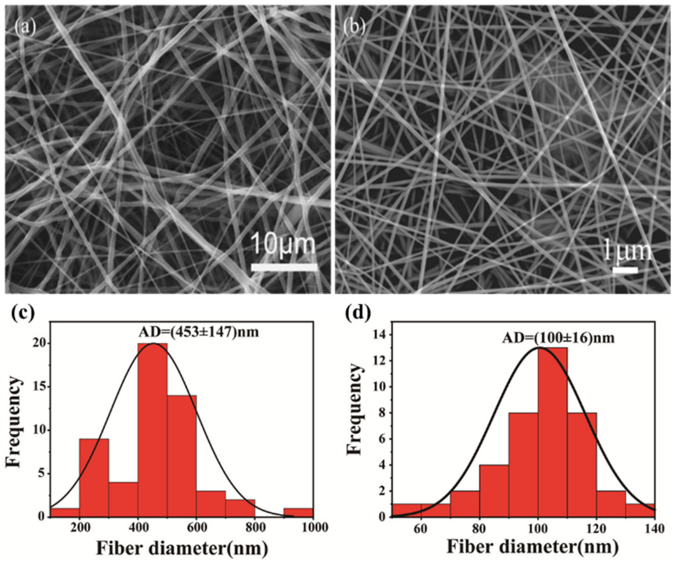

Figure S2. (**a**,**b**) SEM images and (**c**,**d**) corresponding diameter distribution of (**a**,**c**) single PCL and (**b**,**d**) GEL nanofiber mats.

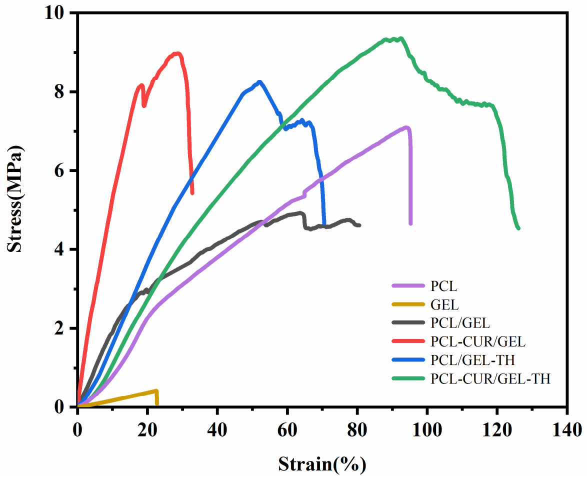

Figure S3. Stress-strain curve of different nanofiber mats in swollen conditions.

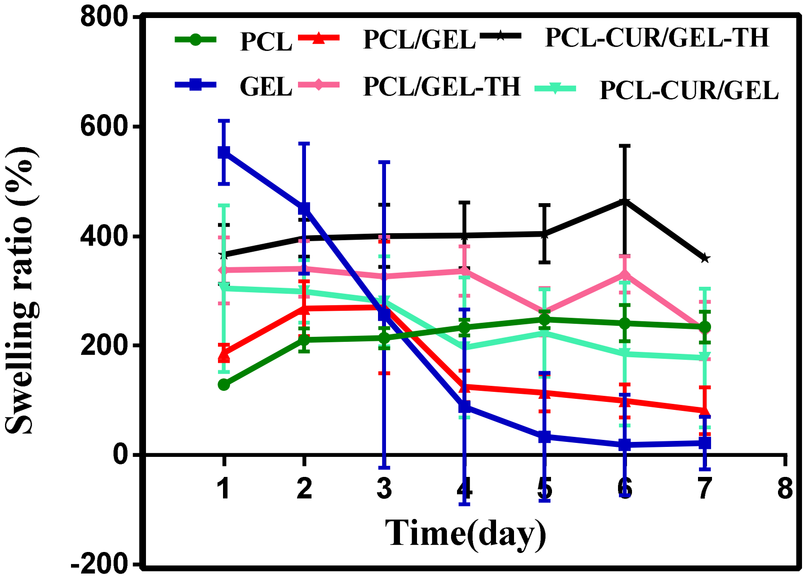

Figure S4. Swelling ratio of different nanofiber mats for 7days.

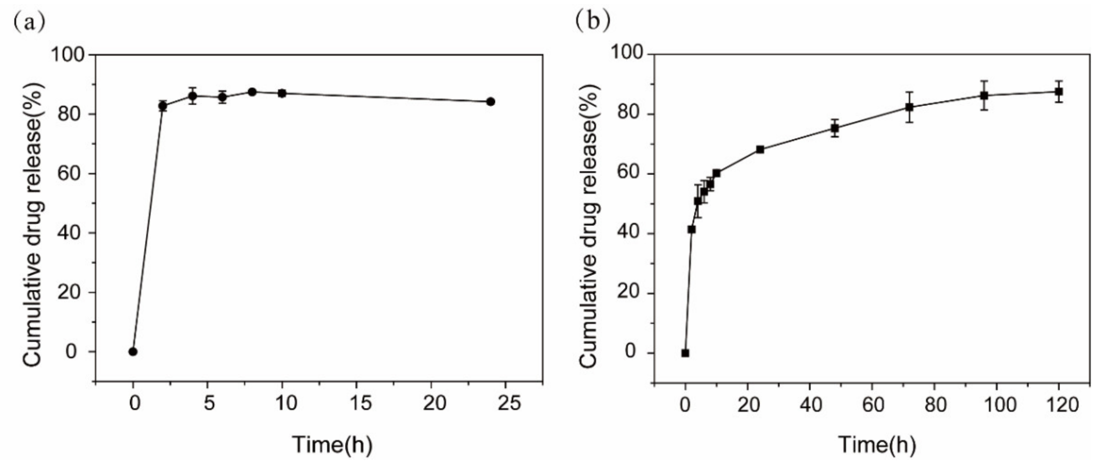

Figure S5. In vitro release profiles of drugs at different time, (**a**) TH; (**b**) Cur.

Tables

Table S1. The wavenumber of the main bands for different samples.

Samples	Wavenumber (cm^{-1})	Functional Group
**PCL**	1240, 1165	C-O-C
	1725	-C=O
**GEL**	1647	Amide I
	1548	Amide II
	2949, 2865	$-CH_2$
**Cur**	1430	C-H
	1510	-C-C
	1600	-C=O
	1277	C-O
	3510	Phenolic hydroxyl group
**TH**	1613	-C=O
	1579	NH_2
**PCL/GEL**	1240, 1165	C-O-C
	1725	-C=O
	1647	Amide I
	1548	Amide II
	2949, 2865	$-CH_2$
**PCL-CUR/GEL**	1240, 1165	C-O-C
	1725	-C=O
	1647	Amide I
	1548	Amide II
	2949, 2865	$-CH_2$
**PCL/GEL-TH**	1240, 1165	C-O-C
	1725	-C=O
	1647	Amide I
	1548	Amide II
	2949, 2865	$-CH_2$
**PCL-CUR/GEL-TH**	1240, 1165	C-O-C
	1725	-C=O
	1647	Amide I
	1548	Amide II
	2949, 2865	$-CH_2$

Table S2. The mechanical parameters of different samples.

Samples	Tensile Strength （MPa）	Elongation at Break （%）	Elastic Modulus (MPa)
GEL	5.59 ± 0.74	13.53 ± 6.40	591.30 ± 24.00
PCL	8.48 ± 3.78	122.22 ± 8.46	74.32 ± 26.59
PCL/GEL	9.16 ± 1.24	39.31 ± 5.98	759.75 ± 79.42
PCL-CUR/GEL	3.04 ± 1.11	41.66 ± 10.14	430.99 ± 81.87
PCL/GEL-TH	12.96 ± 3.51	68.68 ± 32.40	1276.78 ± 136.51
PCL-CUR/GEL-TH	9.58 ± 1.78	139.10 ± 7.12	66.42 ± 9.50

Table S3. Electrospinning parameters for single and coaxial nanofiber mats.

Samples	Needle	Voltage (kV)	Push Speed (mm/min)	Distance between Needle and Receiver (cm)
GEL	21	8-10	0.1	12–15
PCL	21		0.1	
Coaxial electrospinning	Core:21<br>Shell:15		Core:0.02<br>Shell:0.04	
